# Exotic Plant Infestation Is Associated with Decreased Modularity and Increased Numbers of Connectors in Mixed-Grass Prairie Pollination Networks

**DOI:** 10.1371/journal.pone.0155068

**Published:** 2016-05-16

**Authors:** Diane L. Larson, Paul A. Rabie, Sam Droege, Jennifer L. Larson, Milton Haar

**Affiliations:** 1 Northern Prairie Wildlife Research Center, U. S. Geological Survey, St. Paul, Minnesota, United States of America; 2 Western Ecosystems Technology, Inc., Laramie, Wyoming, United States of America; 3 Patuxent Wildlife Research Center, U.S. Geological Survey, Laurel, Maryland, United States of America; 4 Polistes Foundation, St. Paul, Minnesota, United States of America; 5 Badlands National Park, Interior, South Dakota, United States of America; Oregon State University, UNITED STATES

## Abstract

The majority of pollinating insects are generalists whose lifetimes overlap flowering periods of many potentially suitable plant species. Such generality is instrumental in allowing exotic plant species to invade pollination networks. The particulars of how existing networks change in response to an invasive plant over the course of its phenology are not well characterized, but may shed light on the probability of long-term effects on plant-pollinator interactions and the stability of network structure. Here we describe changes in network topology and modular structure of infested and non-infested networks during the flowering season of the generalist non-native flowering plant, *Cirsium arvense* in mixed-grass prairie at Badlands National Park, South Dakota, USA. Objectives were to compare network-level effects of infestation as they propagate over the season in infested and non-infested (with respect to *C*. *arvense*) networks. We characterized plant-pollinator networks on 5 non-infested and 7 infested 1-ha plots during 4 sample periods that collectively covered the length of *C*. *arvense* flowering period. Two other abundantly-flowering invasive plants were present during this time: *Melilotus officinalis* had highly variable floral abundance in both *C*. *arvense-*infested and non-infested plots and *Convolvulus arvensis*, which occurred almost exclusively in infested plots and peaked early in the season. Modularity, including roles of individual species, and network topology were assessed for each sample period as well as in pooled infested and non-infested networks. Differences in modularity and network metrics between infested and non-infested networks were limited to the third and fourth sample periods, during flower senescence of *C*. *arvense* and the other invasive species; generality of pollinators rose concurrently, suggesting rewiring of the network and a lag effect of earlier floral abundance. Modularity was lower and number of connectors higher in infested networks, whether they were assessed in individual sample periods or pooled into infested and non-infested networks over the entire blooming period of *C*. *arvense*. *C*onnectors typically did not reside within the same modules as *C*. *arvense*, suggesting that effects of the other invasive plants may also influence the modularity results, and that effects of infestation extend to co-flowering native plants. We conclude that the presence of abundantly flowering invasive species is associated with greater network stability due to decreased modularity, but whether this is advantageous for the associated native plant-pollinator communities depends on the nature of perturbations they experience.

## Introduction

Invasive plant species can become integrated into plant-pollinator networks and often occupy key roles within those networks [1–4]. Within communities harboring many invasive plants, in particular those providing abundant pollen and nectar, nestedness also may increase [2, 5], thereby making networks more resistant to perturbation. Similarly, invasive flowering plants can reduce modularity, although this may or may not result in enhanced network resilience, depending on the nature of the perturbation [6]. Despite these potentially potent effects of plant invasion, Tiedeken and Stout [7] found considerable structural network resilience when they compared pollinator communities during and after the flowering season of *Rhododendron ponticum*, an abundantly-flowering invasive plant, as did Vilá et al. [8] for variously invaded versus non-invaded sites across Europe.

Because the majority of pollinating insects are generalists [9] and individuals can outlive and overlap the flowering periods of many potentially suitable pollen-producing plant species, they may be quite adept at switching to different host plants during the course of a season. This continuous “rewiring” [10] of network connections not only allows invasive plants to integrate into existing networks, but also may change the temporal pattern of pollination rates of native plants within the network if some pollinators temporarily switch to a more abundant invasive resource [11–13]. Furthermore, invasive flowering plants potentially attract new pollinators to native plants within the community or may out-compete native plants for pollinators, with a variety of possible outcomes for native plant reproduction [14, 15].

Although many aspects of existing native plant network topology seem to withstand invasion of non-native flowering plants (e.g., nestedness, connectance, linkage; but see [2]), network modularity may be an exception because the invasives often become network or module hubs or connectors, thus assuming structurally important roles. When an invasive plant provides an exceptionally rich resource, it may absorb modules that were previously distinct into its own module or increase connection among modules, thereby reducing the likelihood of the network fracturing into vulnerable shards, but also increasing the transmission of perturbations throughout the network if structurally important species are removed [16]. The implications for the resident native plant and pollinator communities thus depend on details of the invasion process [17] and subsequent perturbations [16].

**Fig 1:**
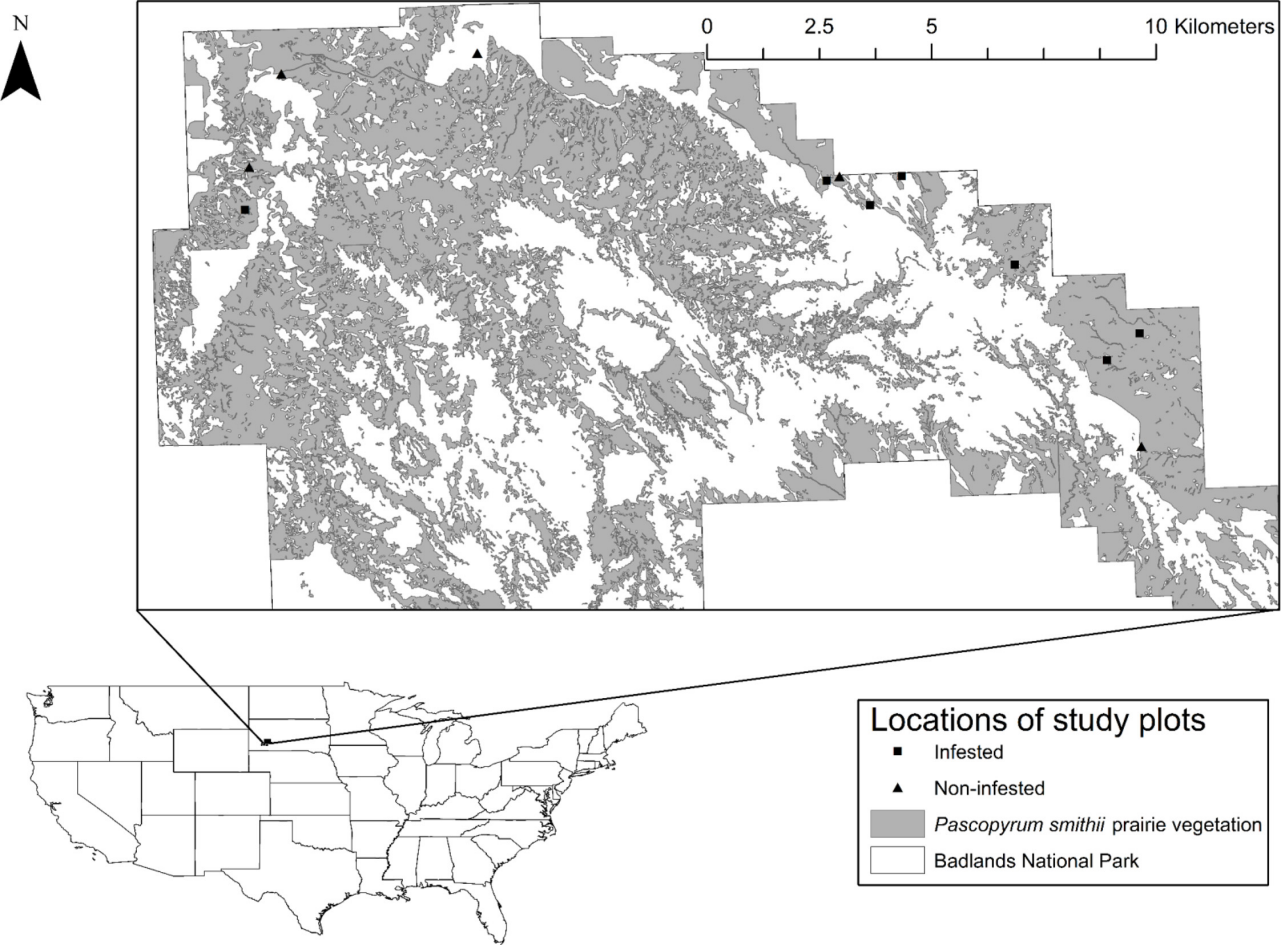
Locations of study sites at Badlands National Park, South Dakota, USA.

In this study, we describe changes in the network topology and modular structure of networks invaded and not invaded by the generalist non-native flowering plant *Cirsium arvense* (L) during its flowering season in mixed-grass prairie at Badlands National Park, South Dakota, USA (Fig 1). The study began at the onset of flowering by *C*. *arvense* and continued through *C*. *arvense* flower senescence, 22 June– 17 July, 2012. Objectives of the study were to compare network-level effects, including modularity, as they propagate over the season, and the fate of networks when the invasive plant is no longer a dominant part of the resource landscape, between networks infested or not infested by *C*. *arvense*. As a serious agricultural weed, *C*. *arvense* control and eradication programs are common, including programs at Badlands National Park, so its effects on plant-pollinator community characteristics and stability are of interest to resource managers as well as to pollination ecologists. Although it is the only invasive plant that is actively managed at our study sites, *C*. *arvense* is not the only abundant invasive plant at these study sites; *Melilotus officinalis* (L.) Lam. and *Convolvulus arvensis* (L.) also present rich resources for pollinators and their effects also are considered.

## Materials and Methods

### Study site

**Fig 2:**
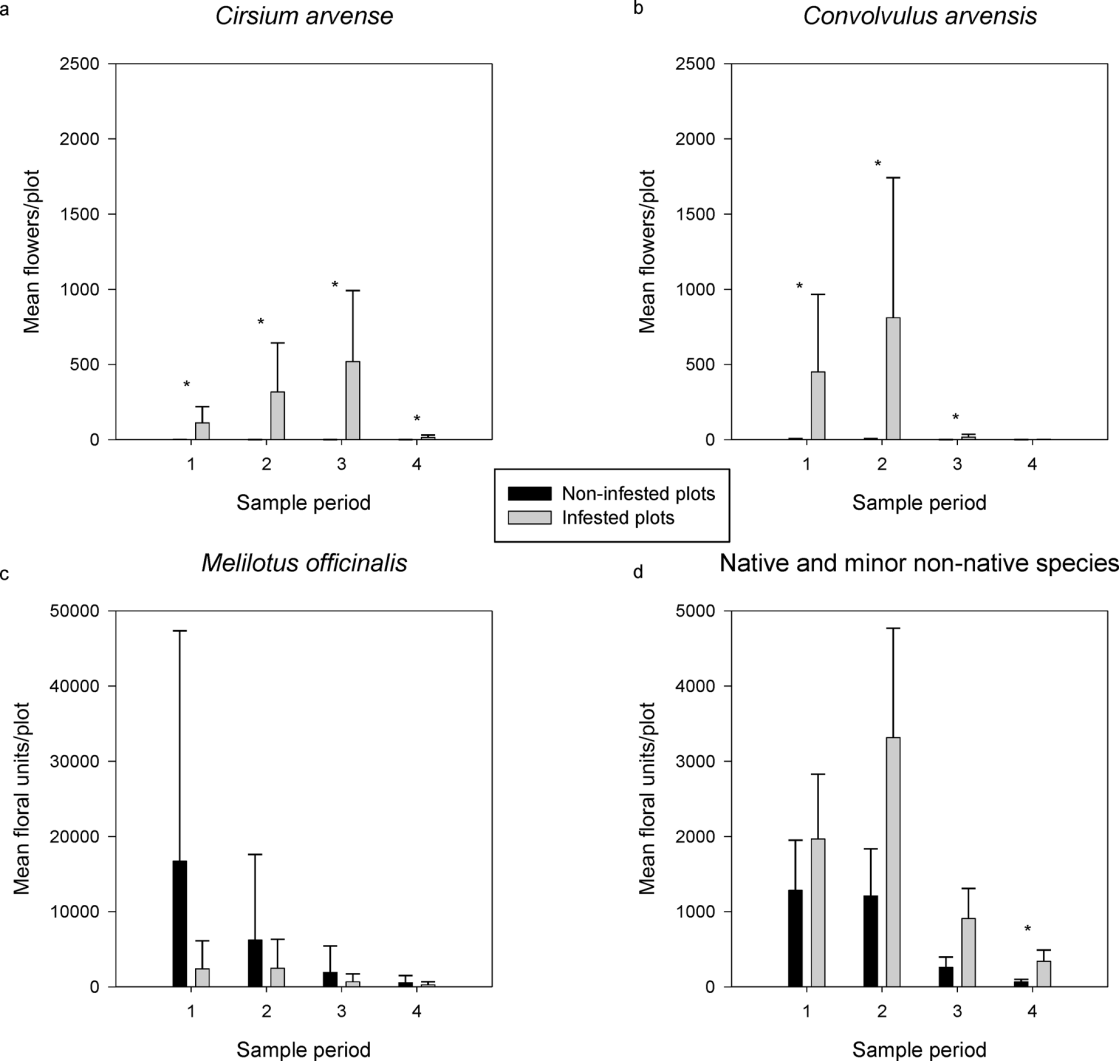
**Mean number of flowers counted per sample period/plot on non-infested and infested plots of (a) *C*. *arvense*, (b) *C*. *arvensis*, (c) *M*. *officinalis*, and (d) native and minor non-native plants.** Shown are least square means and their standard errors. Asterisks denote significant differences between means on infested and non-infested plots within a sample period as determined by Fisher’s Least Significant Difference. Note that the Y-axes differ among some panels.

The study was conducted in western wheatgrass (*Pascopyrum smithii* (Rydb.) Á. Löve) prairie vegetation at Badlands National Park (Fig 1; Table 1) under a permit issued by the park. Study plots were selected from areas known by National Park Service staff to contain or be free of infestation by *C*. *arvense* and subsequent references to infested and non-infested are with reference to *C*. *arvense*, not the other non-native species. Five plots had no or negligible amounts of *C*. *arvense* within 200 m and seven plots contained *C*. *arvense* in varying densities. All plots were ≥ 300 m from any other plot, except for one infested and one non-infested plot that were 225 m from each other at their nearest points. Although it is certainly possible for pollinating insects to fly > 200 m, the likelihood is less for smaller insects and declines as the distance increases [18, 19]. A nonmetric multidimensional scaling analysis of the matrix of plots by insect species captured detected no structure in the insect community (S1 Appendix), which suggests that it is reasonable to treat the plots as replicate samples. The plots were approximately 1-ha, 133 m x 75 m, centered on the *C*. *arvense* population (if present). Ten 2-m x 75-m belt transects, 13 m apart at their mid-points, traversed the plots and were used for flower counts and insect sampling (see below). Sampling began on 22 June and ended on 17 July 2012; no sampling occurred from 30 June until 8 July. The season was divided into 4 sample periods, each typically 4 days long, although the final one extended for 6 days due to unfavorable weather conditions. Total flower counts of native and minor non-native (e.g., *Taraxacum officinale* F.H. Wigg., *Tragopogon dubius* Scop., etc.) species were similar in the infested and non-infested plots (see Results, Fig 2).

**Table 1:** Locations of study plots at Badlands National Park.

Plot ID	Latitude	Longitude
BC1	43.88528	-102.24
BC2	43.87966	-102.249
BC3	43.8849	-102.26
BCC	43.88564	-102.257
BP1	43.88277	-102.422
BP2	43.89129	-102.42
BP3	43.90975	-102.411
BPC	43.91268	-102.356
MB1	43.86681	-102.209
MB2	43.84703	-102.184
MB3	43.85217	-102.175
MBC	43.82944	-102.176

The study was originally conceived as a removal experiment, with 6 infested plots designated for removal, 3 non-removal, and 3 non-infested. At the time of *C*. *arvense* removal, however, unseasonably hot weather resulted in rapid senescence of all *C*. *arvense* flowers, so that removal and non-removal treatments were essentially identical (S1 Table). In addition, *C*. *arvense* plants in 2 of the plots identified as infested failed to produce flowers; these were subsequently categorized as non-infested, resulting in 5 non-infested and 7 infested plots as described above. A nonmetric multidimensional scaling analysis (performed in PCOrd version 6) of the matrix of plots by flower counts accounted for 78% of the variation with 3 axes; the analysis did not separate plots by infestation status (with or without including *C*. *arvense* in the matrix), suggesting that despite invasion, the plant communities had not diverged (S1 Appendix). Therefore, we believe that categorizing plots where *C*. *arvense* failed to flower as non-infested for purposes of pollinator analyses is justified.

### Flower counts

We counted the number of open flowers (or for species with clustered flowers, we counted pre-determined ‘floral units’ such as racemes) by species on each of the 10 transects per plot once or twice during each sample period. Transect width was narrowed to 25 or 50 cm for some species that at times had very abundant flowers (*C*. *arvense*, *Convolvulus arvensis*, *Erigeron strigosus*, *Medicago sativa*, *Pediomelum argophyllum*, and *Symphoricarpos occidentalis*; 37 of 547 counts of individual species used a reduced transect width), but all counts were standardized to 1-m width in data summaries. If two counts of a plot were done during one sample period, the standardized counts were averaged. *Melilotus officinalis* flowers were too dense to count in the same way as the other species. Instead, we clipped and counted the number of racemes present in a sample of rectangles delineated by 0.25 m and the mean length of a pace at low and high *M*. *officinalis* density; the number of paces that intersected *M*. *officinalis* at low or high density on transects was multiplied by the appropriate number to arrive at an estimate of the number of racemes per transect, which were then standardized to 1-m width.

### Flower-visitors

Insects were sampled every-other day at each plot, weather permitting. We used time-constrained searches in which we collected all insects that were in contact with reproductive parts of flowers along the same transects in which flowers were counted, but in this case each transect was 2-m wide. Transects were searched for 20 minutes each (excluding long handling times for some insects) and no more than 5 insects were collected from any individual plant to avoid biasing the sample in favor of more densely flowering species and to ensure that even sparsely-flowering plant species were sampled on each transect. Insect sampling only occurred on days with warm temperatures and low to moderate winds. Insects were captured individually by hand netting and transferred to a vial that had been charged with ethyl acetate. After the insect was quiet in the vial, it was transferred to a glassine envelope that had been labeled with the date, site, and flower species on which the insect had been captured. The envelope was then placed into a larger jar, also charged with ethyl acetate, for transport back to the lab. Vials were cleaned with a tissue after removal of each insect to reduce pollen transfer from one insect to another. After pollen was removed from the insects (see below), the pinned specimens were identified to species or, in some cases, to morphospecies.

### Pollen

In the lab, insects were removed from the envelopes individually and pinned. Pollen was removed from their bodies using small cubes of fuchsin jelly [20]. The jelly was rubbed over all parts of the insect and more than one cube could be used on large insects or in cases in which the pollen load was particularly dense. The cubes were placed on slides, heated gently until melted, covered with a coverslip, and a thin strip of latex paint was applied around the edges of the coverslip to seal it. Work surfaces and forceps were wiped clean after each insect was processed.

Pollen was identified under a light microscope at 10 – 100x with the aid of a reference collection made in and around the study plots. The number of pollen grains per species was counted. Fewer than 10 pollen grains of a flower species was considered contamination [21]. Ten or more pollen grains were considered evidence of a visit to that flower species. Slides were searched systematically and exhaustively for pollen species and all were recorded; it was not until data analysis that species represented by < 10 grains were removed from the data set. We attempted to limit our analyses to legitimate pollinators. From the data set comprising insects and their pollen loads, we removed any insect species, with the exception of bees, that never carried ≥10 pollen grains of any flower species.

### Data analysis

We compared the mean number of pollinator species that visited native plants and the mean number of interactions (i.e., insect species interacting with plant species) involving native plants during each sample period on infested and non-infested plots (i.e., the model included an interaction term for sample period x infestation status) with the GLIMMIX Procedure in SAS 9.4. Total flower counts (ln-transformed to reduce skewness) were used as covariates in the models; we tested for common slopes as described in Milliken and Johnson [22] and found no difference, so proceeded to test the interactions of each term in the model with the covariate, removing the highest-order interaction in term when it was nonsignificant. Plot nested within infestation status was specified as a random effect and a negative binomial distribution was used for both comparisons. Post-hoc comparisons were made with Fisher’s Least Significant Difference. [22].

Bipartite networks were constructed using both the species the insect was captured on and the pollen it carried as evidence of visitation. Note that an individual insect could participate in > 1 interaction if the species of pollen it carried differed from the flower species upon which it was captured, but we could not distinguish if conspecific pollen derived from the flower on which the insect was captured, nor could we determine how many visits to different conspecific flowers each pollen species on an insect’s body represented. Data were pooled within four sample periods (22–25 and 26–28 June; 9–12 and 13–17 July) to construct one infested and one non-infested network per period. The R package, Bipartite [23], and methods in Dormann [24] were used to calculate the whole-network metrics weighted NODF (a measure of nestedness), Shannon diversity and evenness of interactions, H2 (a measure of overall network specialization) and connectance (proportion of realized links in a network). One species-level metric was calculated: the weighted mean effective number of interaction partners; this is termed vulnerability for plants and generality for insects.

The difference between metrics for infested and non-infested networks was calculated within each sample period. We did not make comparisons across sample periods because the influence of phenology is likely inseparable from infestation effects. A permutation test was used to assess the magnitudes of differences between infested and non-infested networks. For each permutation, five plots were randomly selected to construct a ‘non-infested’ network, the remaining seven plots were used to construct a random ‘infested’ network, and the difference between them was calculated. Our permutations used the same number of plots for infested and non-infested networks as were used to calculate the observed metrics because some of the metrics are sensitive to network size. One thousand permutations were assembled, resulting in a null population of 1000 differences in network metrics. The observed difference in network metrics was considered significant if it was greater or less than the central 95% of random differences.

For the modularity analysis, plots were pooled over sample period to construct one infested and one non-infested network per sample period. Because modularity is sensitive to sampling intensity [25], two infested plots were randomly removed before assembling the networks, leaving 5 plots each in infested and non-infested networks. Modularity of each network was calculated using the Netcarto software package [26]. We calculated 95% bounds for the distribution of modularity from 100 randomized networks to test whether the observed value differed from that of a random network. Roles were assigned to species as described by Dupont and Olesen [27, 28] based on participation coefficient (a measure of connections outside a node’s module) and within-module relative degree (a measure of connections within a node’s module; [26, 29]). Pooling of plots for the modularity analysis precluded replication in the data set; to reduce the possibility that role distributions were influenced by outlier plots, each plot was removed in turn from the data set and participation coefficients and within-module relative degrees recalculated with the 4 remaining plots within infestation categories. Because the limits we set for sample periods were biologically arbitrary (as opposed to the entire season, which was set to encompass the flowering season of *C*. *arvense*), we also pooled infested sites into one modularity analysis and non-infested sites into a separate modularity analysis, to assess effects of infestation over the entire season. Methods follow those described above.

## Results

*Cirsium arvense* flower counts were significantly higher in infested than non-infested plots (F_1,10_ = 26.25, P = 0.0004), per the study’s design, but the high standard errors resulted in no significant differences among sample periods (F_3,30_ = 1.45, P = 0.2472; Fig 2A). *Convolvulus arvensis* had a significant infestation x sample period interaction (F_1,10_ = 5.94, P = 0.0026; Fig 2B); it flowered more abundantly in infested plots than non-infested in the first three sample periods, but had declined to near zero flowers by the final sample period. High variance in flower abundance among plots resulted in no significant differences in *M*. *officinalis* flower counts between infestation statuses (F_1,10_ = .25, P = .6301) but there was a significant difference among sample periods (F_3,30_ = 11.97, P<0.0001; Fig 2C); flowering declined over time. Counts of native flowers (including minor non-native species) did not vary significantly between infested and non-infested plots (F_1,10_ = 3.14, P = 0.1067) but did vary among sample periods (F = 47.07, P<0.0001; Fig 2D). Interestingly, when all floral counts were combined in a single analysis, means differed by sample period (F_3,30_ = 21.03, P<0.0001), being stable for the first two sample periods and declining in the third and fourth, but did not differ by infestation status (F_1,10_ = 0.38, P = 0.5503) and there was no interaction (F_3,30_ = 0.02, P = 0.9959; S1 Fig).

**Fig 3:**
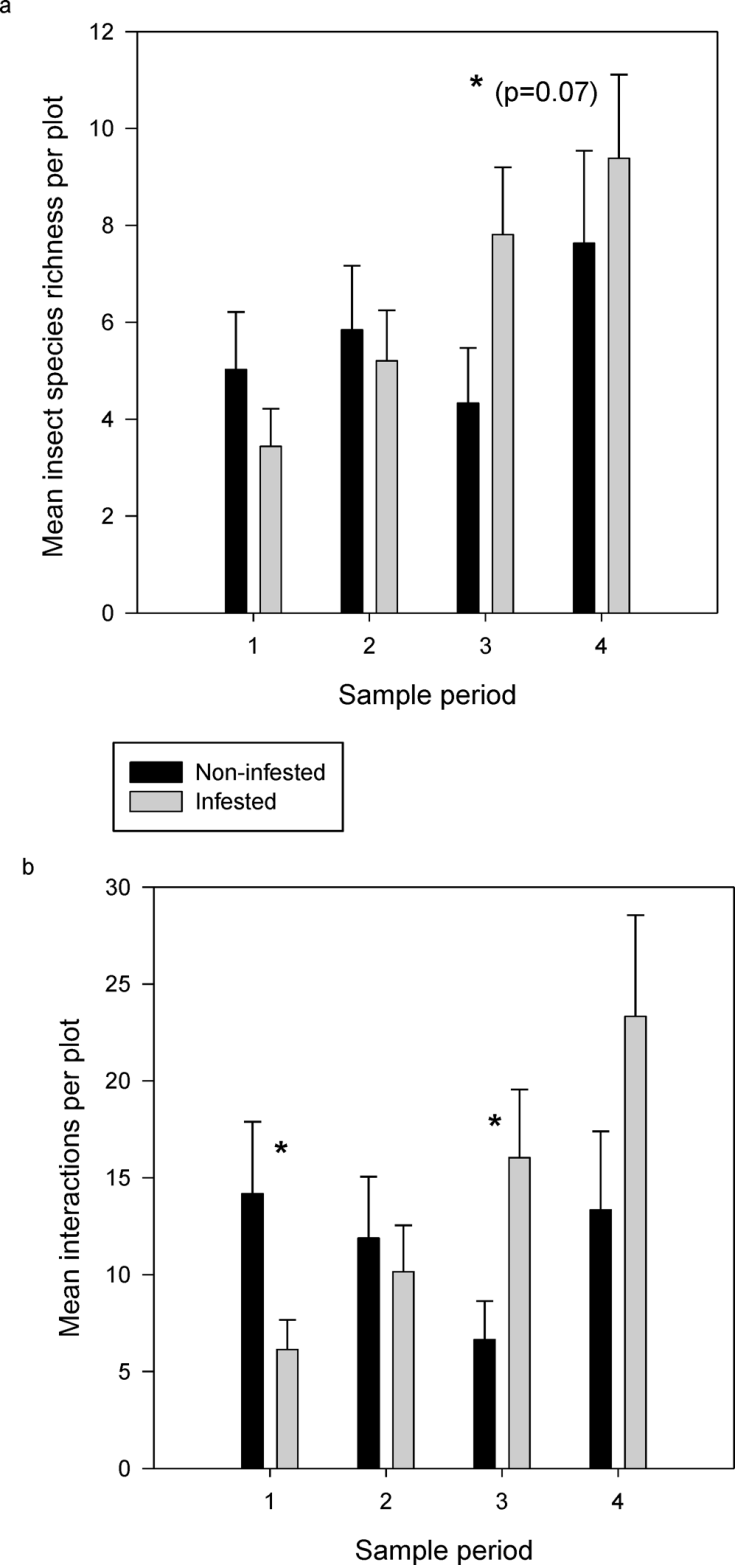
**Comparison of (a) mean number of insect species visiting native plant flowers and (b) mean number of interactions/plot involving native (and minor exotic) plants on infested and non-infested plots over four sample periods.** Shown are least-square means and standard error of the means. The asterisks indicate a significant difference between means during that sample period as determined by Fisher’s Least Significant Difference.

In all, we recorded 490 plant-pollinator interactions in the 5 non-infested plots (126 included one of the three focal invasive species), 1195 in the 5 infested plots (558 included one of the three focal invasive species) used for the modularity analysis, and 1570 in all 7 infested plots. Recall that infestation is with reference only to *C*. *arvense*. In the modularity data set, 109 insect and 36 plant species occurred in the infested networks and 90 and 31, respectively, in the non-infested. Considering plants and insects together, 36% occurred only in infested plots, 22% only in non-infested plots, and 42% in both. The number of pollinator species that visited native (including minor exotic) plants did not vary between infested and non-infested plots (F_1,10_ = 0.14, P = 0.7192; Fig 3A) although species richness was marginally lower at non-infested plots in the third sample period (T_29_ = -1.82, P = 0.074). Number of interactions that involved native and minor exotic plants was higher in non-infested plots in the first sample period (T_29_ = 2.36, P = 0.0259; Fig 3B), but declined steadily until it was significantly lower in non-infested plots in the third sample period (T_29_ = -2.42, P = 0.0244) before rebounding in the fourth sample period. The covariate, natural log of total flower counts, was a significant main effect in both models (F_1,29_ = 11.99, P = 0.0017 and F_1,29_ = 10.94, P = 0.0029 for insect species richness and number of interactions, respectively) but it had no significant interactions with model terms. Significant terms in the overall models were sample period (F_3,29_ = 3.26, P = .0356) for insect species richness and the sample period x infestation interaction (F_3,29_ = 5.30, P = 0.0049) for number of interactions.

### Network-level measures

**Fig 4:**
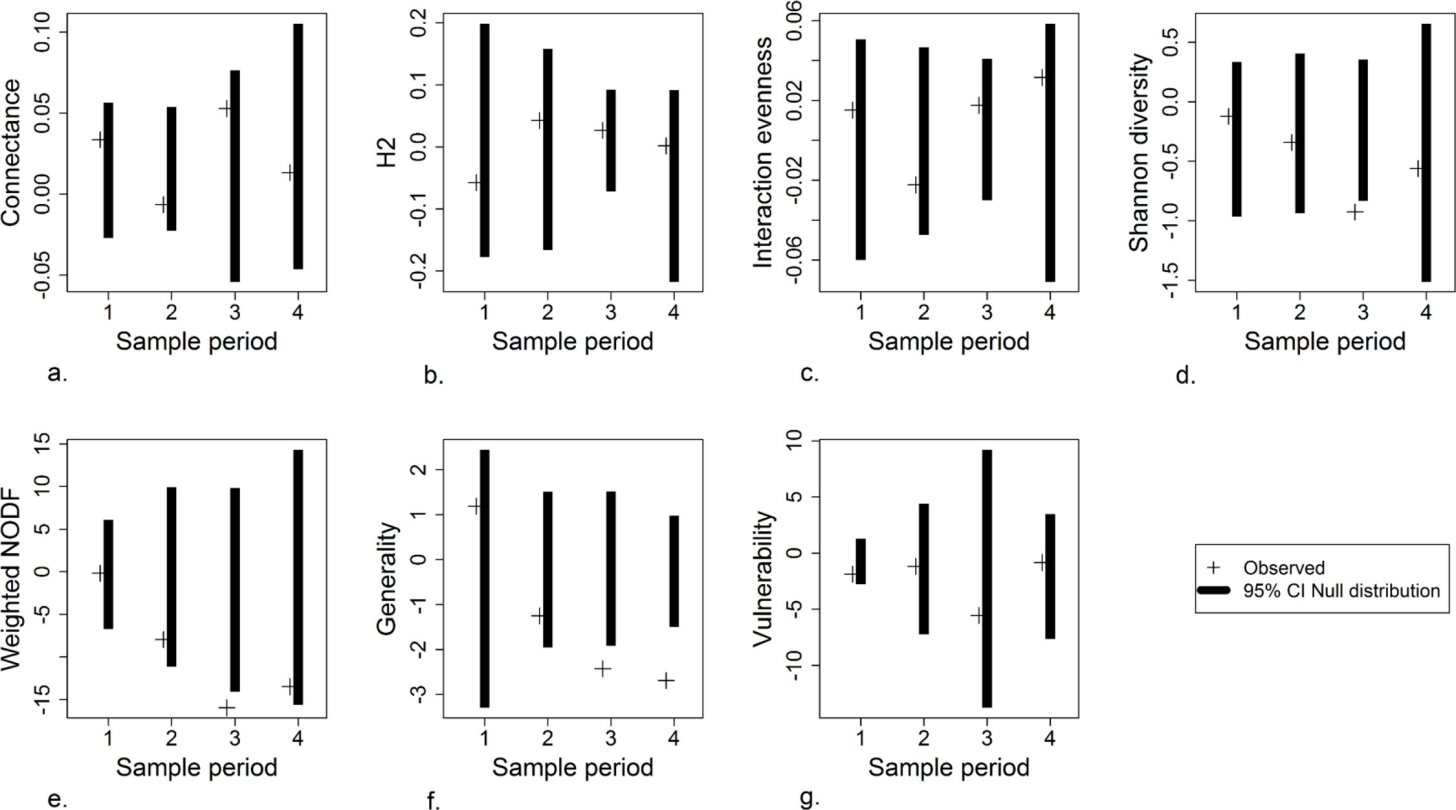
Network metrics. Each panel shows the observed difference in the metric (non-infested–infested, so negative numbers indicate that values in infested networks were higher than in non-infested networks) and the 95% confidence interval from the permutation test for each sample period.

Most of the network level properties we measured did not differ between infested and non-infested networks, but when differences occurred, they were always in the third or fourth sample period (Fig 4). For example, weighted NODF (Fig 4E) for infested and non-infested networks were similarly nested at the first sample period, but infested networks became more nested as *C*. *arvense* and *C*. *arvensis* flowers increased (and *M*. *officinalis* flowers declined); the difference was significant only in the third sample period, however, which was at the peak of *C*. *arvense* flower abundance, but past the peak of *C*. *arvensis* (Fig 2). Shannon interaction diversity (Fig 4D) followed a similar pattern. Generality, referring to the variety of resources used by the pollinators, was greater in infested than non-infested networks in the third and fourth sample periods (Fig 4F).

### Modularity

**Fig 5:**
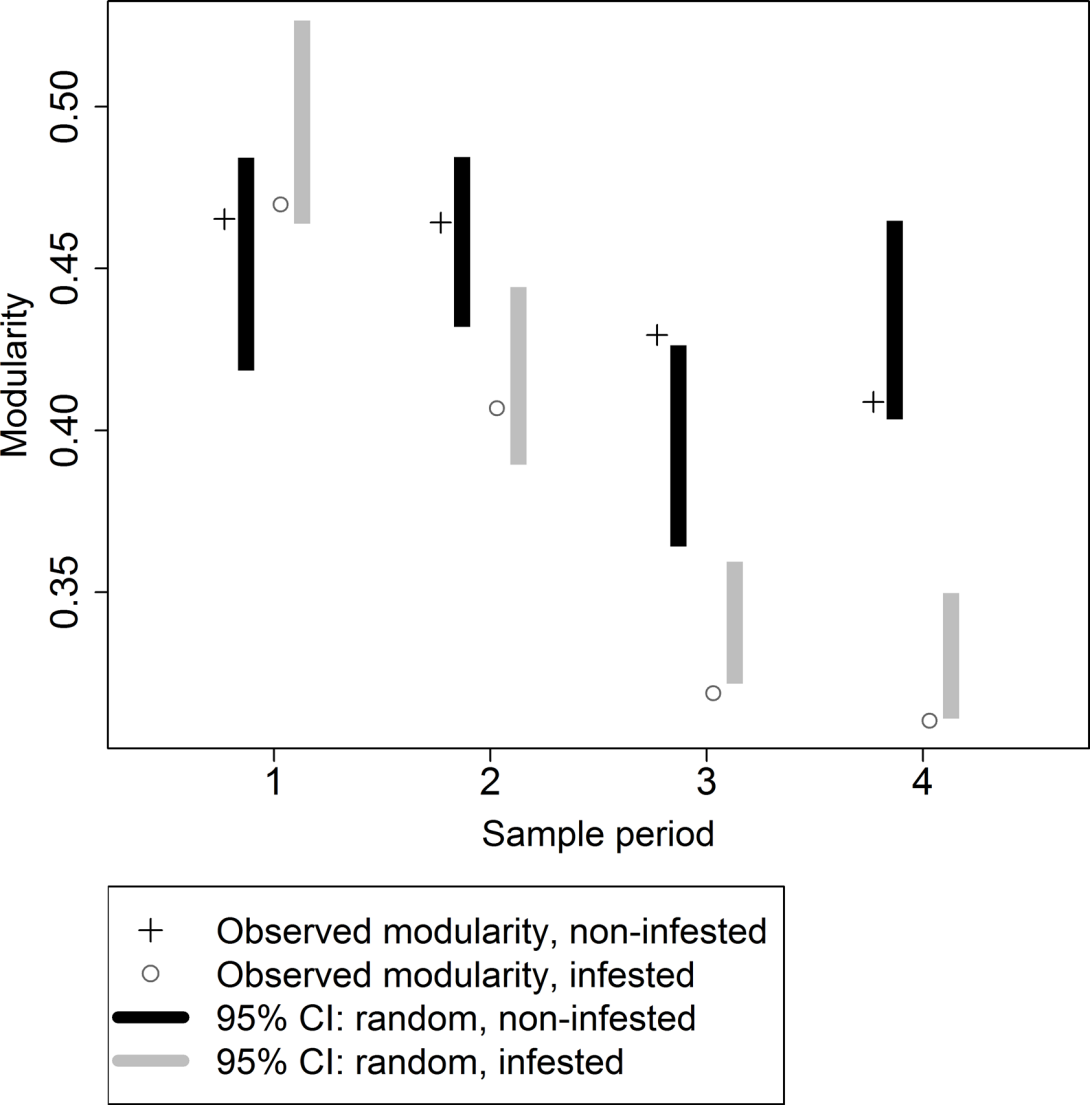
Observed modularity and 95% confidence interval from randomly assembled networks for each sample period on infested and non-infested plots.

Because modularity depends on network size, modularity of infested and non-infested networks cannot be directly compared. Rather, the modularity of an observed network can be evaluated with respect to the distribution of modularity from random networks [26]. The modularity of non-infested networks was closer to the upper end of the 95% confidence intervals for random networks for the first two sample periods, but significantly lower than that of a random network during the final two sample periods (Fig 5). The modularity of infested networks was closer to the lower end of the 95% confidence intervals for random networks during all sample periods, significantly so during the third and fourth (Fig 5). Modularity of non-infested networks differed from the random networks only in the third sample period, when modularity was higher than in the random networks. There were 8, 6, 6, and 5 modules in infested networks during sample periods 1–4, respectively, and 5, 6, 5, and 6 modules in non-infested networks. Complete results for the modularity analysis by sample periods can be found in S2 Table.

**Fig 6:**
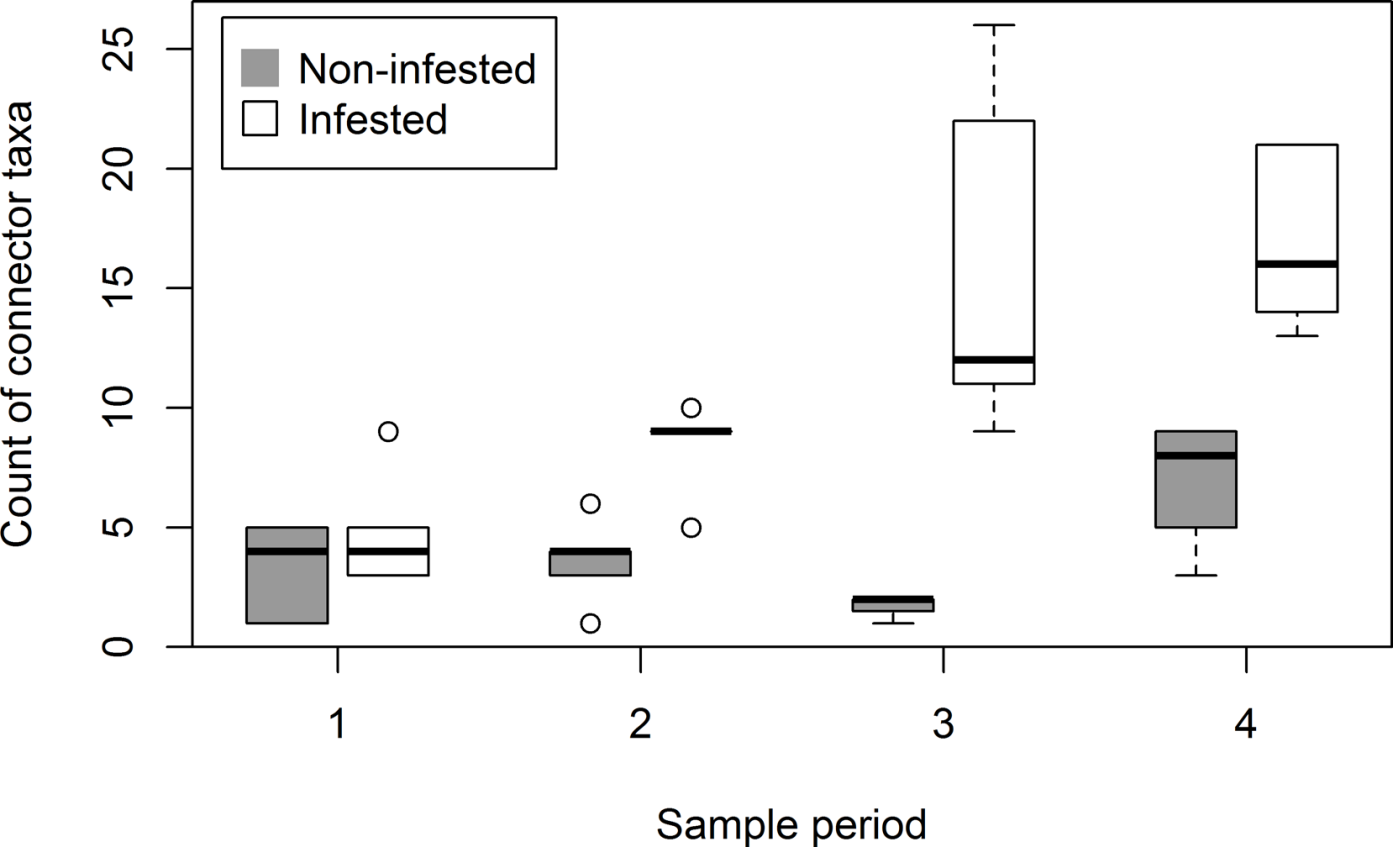
Boxplots showing distributions of counts of connector taxa at each sample period in infested and non-infested plots. Distributions were generated using a jack-knife recalculation of the modularity analysis. Boxes capture the first and third quartiles of the distribution, whiskers capture data points within ±1.5 times the interquartile range, and means are shown as horizontal bars through the boxes.

The most striking difference between infested and non-infested networks was the distribution of roles, especially connectors (Table 2). This difference was robust to removal of individual plots, so is unlikely to have been caused by an outlier (Fig 6). When plots were pooled over sample periods into infested and non-infested groups for analysis, 32% of nodes in the modules in infested plots were connectors compared to 21% in modules in non-infested plots, which doubtless contributed to the lower modularity in infested networks. For the pooled analysis, observed modularity in non-infested network was significantly greater than randomly assembled networks (observed = 0.398858, random = 0.372055, sigma_rancom_ = 0.006635) and in the infested network was significantly lower than randomly assembled networks (observed = 0.31628, random = 0.33156, sigma_random_ = 0.006688.

**Table 2:** Modularity summary: number of taxa (plant plus insect) in each of 3 roles for 5 infested and 5 non-infested plots. Only insect taxa documented to be pollen carriers were included in the analysis.

		Infested plots Role			Non-infested plots Role		
Sample period	Module hub^1^	Connector	Hub	Peripheral	Connector	Hub	Peripheral
**1**	*C*. *arvense*	1	1	11			
**1**	*Medicago sativa*	1	1	8			
**1**	*None*	3		27	6		42
**Subtotal (proportion)**		5 (0.094)	2 (0.038)	46 (0.87)	6 (0.125)		42 (0.875)
**2**	*C*. *arvense*		1	12			
**2**	*Erigeron strigosus*				2	1	9
**2**	*M*. *officinalis*	1	1	12			
**2**	*M*. *officinalis/S*. *occidentalis*				1	2	19
**2**	*None*	5		13	1		18
**2**	*R*. *columnifera*					1	12
**2**	*S*. *occidentalis*	4	1	10			
**Subtotal (proportion)**		10 (0.17)	3 (0.05)	47 (0.78)	4 (0.06)	4 (0.06)	58 (0.88)
**3**	*C*. *arvense/M*. *officinalis*	1	2	26			
**3**	*Euphorbia marginata*	8	1	8			
**3**	*Helianthus annuus*				2	1	7
**3**	*M*. *officinalis/S*. *occidentalis*					2	19
**3**	*None*	2		14	1		4
**3**	*R*. *columnifera*					1	10
**3**	*R*. *columnifera/S*. *occidentalis*	4	2	14			
**Subtotal (proportion)**		15 (0.18)	5 (0.061)	62 (0.76)	3 (0.064)	4 (0.085)	40 (0.85)
**4**	*C*. *arvense*	4	1	5			
**4**	*Cirsium flodmanii*	6	1	9			
**4**	*E*. *marginata*	3	1	9			
**4**	*M*. *officinalis*				3	1	6
**4**	*M*. *officinalis/S*. *occidentalis*	1	2	17			
**4**	*Melissodes BADL10*	4	1	9			
**4**	*None*				7		22
**4**	*S*. *occidentalis*					1	12
**Subtotal (proportion)**		18 (0.25)	6 (0.082)	49 (0.67)	10 (0.19)	2 (0.038)	40 (0.78)
**Single analysis**^**2**^ **(proportion)**		**35 (0.32)**	**8 (0.055)**	**102 (0.70)**	**26 (0.21)**	**5 (0.041)**	**90 (0.74)**

^1^ Collections of peripheral or peripheral and connector species were sometimes identified as modules. When no module hub was identified for a module, “None” is entered. “None” may include more than one module in any sample period/infestation combination.

^2^ Sum of interactions associated with each role when all infested plots were analyzed in one modularity analysis and all non-infested plots were analyzed in one modularity analysis.

The total number of nodes identified as connectors increased from 5 in the first sample period to 15 each in the third and fourth in the infested networks; connectors declined from 6 in the first to 3 in the third, before rebounding to 10 in the fourth sample period in non-infested networks (Table 2). Only three times was the same species a connector in both infested and non-infested networks (when data were analyzed by sample period; S2 Table): a sweatbee *Lasioglossum semicaeruleum*, a bumble bee (*Bombus nevadensis*) and *M*. *officinalis*. Not surprisingly, since plots were centered on it, *C*. *arvense* was a hub species during each sample period in the infested networks, including the fourth, when its abundance in the plots had declined precipitously (Fig 2); interactions in the fourth sample period could be attributed in part to pollen carried by insects visiting the plots. Surprisingly, *C*. *arvensis*, despite its abundance in the earlier sample periods, was never a hub or a connector species when the data were analyzed by sample period; it was a hub species in the pooled infested network, however. *Melilotus officinalis* was a hub in the second, third and fourth periods in both infested and non-infested networks. *Ratibida columnifera*, *Symphoricarpos occidentalis*, and *M*. *officinalis* were the only three species that were hubs in both infested and non-infested networks, whether or not the data were pooled over all sample periods.

The preponderance of connectors in the infested networks was also reflected in individual modules. All three species that occupied hub positions in both infested and non-infested networks had fewer connectors in their non-infested modules (Table 2). Surprisingly, connectors were rarely members of a module with *C*. *arvense* as a hub (Table 2). Instead, they were concentrated in modules that lacked hubs altogether (“None” in the “Module hub” column of Table 2) or with native hubs *Euphorbia marginata* and *Cirsium flodmanii*. However, connectors generally were likely to interact with one of the three focal invasive species: connector insects interacted with *C*. *arvense* 135 times, with *C*. *arvensis* 69 times, and with *M*. *officinalis* 151 times, compared with 438 interactions with all other plant species combined (45% of interactions were with the three focal invasive species; 37% of peripheral insect interactions were with the focal invasive species, summed over all four sample periods).

## Discussion

The presence of abundantly flowering invasive plant species was associated with strong and persistent differences in modularity and the role composition of modules in these northern Great Plains wheatgrass prairie networks. Notably, differences in modularity between infested and non-infested plots were not evident at the first sample period, when *C*. *arvense* was just beginning to bloom but *Convolvulus arvensis* was nearing its peak and *M*. *officinalis* flowers were also abundant. The strongest signals for effects of infestation (with respect to plots centered either on *C*. *arvense* or not) appeared in the third and fourth sample periods for both modularity and network metrics.

Resource abundance, as measured by mean flower counts per plot, tended to be somewhat higher (though the differences were not statistically significant due to high variation) in the infested plots. Generality of pollinator diets was significantly lower in non-infested than infested plots at the third and fourth sample periods, suggesting that as flowering of the three abundant invasive plants declined, rather than leaving the plots, pollinators were switching to native plant species, though these too were declining. The steady increase in interactions involving native plants on infested plots–even in the fourth sample period when invasive species flowering had collapsed–further supports the idea that rewiring was occurring within the infested networks; the greater number of pollinators present in the infested plots (as indicated by interaction frequency) and thus available to visit native species can be considered a lag-effect of infestation. Similarly, in a study of non-native *Rhododendron ponticum*, generality increased in infested study sites after the invasive plant ceased flowering [7], suggesting that insects that had been visiting the invasive plant broadened their diets to other species.

The proportion of insect species that occurred only in the infested plots may therefore represent generalists that were drawn to high floral abundance, but visited other flowers after senescence of the invasives. Such generalists often assume the role of connector in modular networks [30]. The proportion of connectors in our infested and non-infested networks (pooled over sample periods, 32 and 21% respectively) were higher than those reported in other studies (17% and 11% in [6, 31], respectively). Within sample periods, however, the proportion of connectors was similar to that in the invaded networks in the Galapagos [6] while the proportions in our non-infested networks were substantially lower (6%) in the second and third sample periods. A summary calculated from S1 Table of Larson et al. [1], a modularity analysis of data collected over approximately one month in sparsely vegetated areas of Badlands National Park in which connectors were 12 and 14% of species in 2011 and 2010, respectively, suggests that the elevated proportion of connectors found in the current study are not typical of this geographic area of the North American Great Plains more generally. The sites studied in Larson et al. [1] were invaded by *M*. *officinalis* (most abundant in 2010) and *Salsola tragus* (most abundant in 2011), but not by *C*. *arvense* or *C*. *arvensis*. If the proportions reported in the literature and that we calculated from the previous study at Badlands National Park can be considered typical, then it appears that one or more of the invasive species in the current study are affecting networks in opposite ways in infested (increased connectors) and non-infested (decreased connectors) plots when modularity is assessed within sample periods. If plots were close enough to each other that insects could be drawn from non-infested to infested, this could account for that effect; although some of our plots would fall into that category, it seems unlikely to be the total explanation given that three of the non-infested plots in the western portion of the park were quite isolated from infested plots. A second, related explanation, is that we did not account for all the populations of the invasive plants that occurred outside the plots; insects may be drawn to infestations outside our 200-m buffer around the plots. The insect connectors in the infested plots were not sufficiently associated with *C*. *arvense* to fall within the same module; most were not associated with any hub species and therefore may represent generalists with little allegiance to any plant species.

A second, more intractable, question is raised by the difference in the proportion of connectors in the pooled vs separated sample period analyses. Unlike Dupont and Olesen [27], who found little temporal change in the role composition of heathland networks, our modular structure was variable both over time and between infested and non-infested networks. It is intuitively sensible that smaller samples (e.g., within sample periods) would have fewer actors in each of the roles, so it is not surprising that we found fewer individuals within sample periods. On the other hand, species that occurred in >1 sample period could take on only one role in the pooled analysis, but could occupy the same or a different role in each sample period in which they occur, thus inflating the total pool of species in roles in the sample periods relative to the pooled analyses. This may explain the relatively high proportion of connectors in the pooled analyses compared with the sample-period data, but also calls into question the circumstances under which it is appropriate to compare role proportions between studies.

High levels of modularity in which modules are only sparsely connected to each other can make isolated modules more vulnerable to stochastic events [32], as they lack the potential for a “rescue” effect of connector species whose fates are not tied to that of a single module [1]. On the other hand, declining modularity could be seen to allow greater transmission of perturbation throughout the network, thus destabilizing it [33]. An optimum level of modularity may exist, perhaps varying with abiotic harshness or stochasticity, but our single-year study lacked an appropriate response variable to assess stability independently. Nonetheless, as Tylianakis et al. [33] point out, when a super-generalist invasive species has invaded a network, stability may not be desirable. Olesen et al. [31] reported that on average only 15% of all species in the networks they examined were structurally important to their modules (i.e., were hubs or connectors). Regardless of how the data were analyzed (pooled or by individual sample period) infested networks in the current study always had a higher proportion of structurally important nodes than did non-infested networks, although the differences were very small in the first and fourth sample periods. This suggests that the structure of the infested networks is likely more stable than that of the non-infested networks, particularly at the height of invasive flower abundance. On the other hand, removal of the invasive species may cause unintended consequences for native species in networks with reduced modularity [2, 34], although the rewiring we observed as invasive flowers senesced suggests that may not be the case in this system; on the contrary, invasive plants may be improving pollination of native plants in the vicinity by attracting more pollinators. Because *C*. *arvense* and *C*. *arvensis* are on noxious weed lists in many areas worldwide [35] and managers actively attempt to eradicate them, the implications for the surrounding network are of practical importance.

Our results suggest that infestation has effects that go beyond interactions with the invader itself. *Ratibida columnifera* and *S*. *occidentalis*, for example, were hubs in both infested and non-infested networks, and interacted with more connector species in the former than the latter. Larson et al. [1] found that pollen loads on connector insect species tended to contain more pollen species than did those of peripheral insects, implying the possibility that heterospecific pollen transfer might increase with increased connectors.

As others [2, 36] have found, infested networks in this study were more nested than non-infested networks, but the difference was only significant in the third sample period. In fact, nestedness was virtually identical in the first sample period, at the beginning of *C*. *arvense* bloom but near the peak of *C*. *arvensis* and *M*. *officinalis* bloom. Campbell et al. [5] suggested that invasion by a generalist should increase nestedness, an idea that found support in Albrecht et al. [16]. Most of the native flowering plants on our study plots are in the Asteraceae, as is the focal invasive species, *C*. *arvense*. This family is characterized by generalized, radially symmetric flowers that are easily accessed by most pollinators in the grassland community at Badlands National Park, which may have predisposed them to forage on *C*. *arvense*. In contrast, *M*. *officinalis*, an invasive legume at these study sites, has a more complex flower than those in the Asteraceae. Yet, it too had become integrated into the pollination networks both in these wheatgrass prairie sites and in adjacent sparsely vegetated clay soils of the Badlands sparse vegetation type [1]. Although nestedness is generally considered to lend stability to mutualistic networks, Campbell et al. [37] suggested that over-reliance on a single species may instead prove detrimental to network stability. As noted above, the management goal of extirpating a noxious invader such as *C*. *arvense* should be carefully evaluated with respect to the pollinators that rely upon it.

## Conclusion

Changes in two important aspects of pollination networks, modularity (and related role composition) and nestedness, were associated with infestation by *C*. *arvense*, *C*. *arvensis* and possibly *M*. *officinalis*, though its floral abundance did not follow the same trend as the other two invasive species. Both declining modularity and increasing nestedness result in networks that are resistant to fracture: low modularity by increasing connections and reducing the separation of subsets of communities that interact more with each other than with those outside their modules; and nestedness by increasing dependence of community members on the same subset of highly connected generalists. A novel finding from this study is that although there were many more connectors in infested networks, they did not reside in the same module as *C*. *arvense* during most of its flowering period. Thus, the generalist pollinators apparently attracted by the invasive species were interacting as much if not more with other species in the network. Without a detailed examination of pollen loads received by native stigmas, the effect of these generalist pollinators on native plant populations is unclear [38].

This study was not designed to test differences in effects among the three dominant invasive species; we can make only qualitative observations about their relative influence on the results we observed. *Convolvulus arvensis* flowering peaked early in the season and was much higher in infested than non-infested plots, but as we note above, modularity and nestedness did not vary between infested and non-infested networks at that time. On the other hand, *M*. *officinalis* was often a hub species in both infested and non-infested networks, but reached its peak abundance during the first time period and declined steadily, if non-signficantly, after that. It also presented similar abundances of flowers in infested and non-infested plots. Given that pollinator richness and interactions with native plant species did not change until the last two time periods, when they peaked in infested plots, we feel the evidence more strongly favors the importance of *C*. *arvense* in driving the observed patterns. We note, however, that plots were selected with respect to *C*. *arvense* infestation and results may have been different had we focused on these other non-native species.

Finally, *C*. *arvense* populations are not new to the U.S. Great Plains, or indeed to Badlands National Park and adaptation by both the pollinator and the native plant communities may well have occurred during the time it has been resident. Nonetheless, the similarity of the infested and non-infested networks in the first sample period suggests that changes between the two as flowering progressed were linked to presence of at least *C*. *arvense*, if not some synergistic response to all three invasives. Although implications of reduced modularity for plant-pollinator community stability are somewhat unclear, the evidence for rewiring of interactions as *C*. *arvense* declined suggests that fragmentation of the overall network can be averted, even if *C*. *arvense* is controlled.

## Supporting Information

S1 Appendix Nonmetric multidimensional scaling analyses for flower and insect communities on infested and non-infested plots. Flower species did not separate with respect to *C*. *arvense* infestation. No structure was identified in insect communities.
(DOCX)

S1 Fig Mean number of flowers of all species counted per sample period on non-infested and infested plots. Shown are least square means and their standard errors. Differences between infested and non-infested plots were not statistically significant at any sample period.
(DOCX)

S1 Table Mean *C*. *arvense* flower counts over the four sampling periods on non-removal and removal plots. Thistle flowers were removed between the third and fourth sample periods on the removal plots, but flowers had senesced on the non-removal plots concurrently, so the treatment could not be compared with nontreatment. In addition, some plots that appeared to be infested at the beginning of the study largely failed to flower, further disrupting the planned design.
(DOCX)

S2 Table Results of modularity analyses by sample period for infested and non-infested networks at Badlands National Park. Shown are taxon roles and affiliations with modules, degree, participation coefficient, and within-module relative degree. Results were obtained with the Netcarto program; see manuscript methods for full citation.
(XLSX)

## References

[1] Larson DL, Droege PA, Rabie PA, Larson JL, Devalez J, Haar M, et al Using a network modularity analysis to inform management of a rare endemic plant in the northern Great Plains, USA. Journal of Applied Ecology. 2014;51:1024–32.

[2] Bartomeus I, Vilá M, Santamaria L. Contrasting effects of invasive plants in plant-pollinator networks. Oecologia. 2008;155(4):761–70. 10.1007/s00442-007-0946-1 18188603

[3] Traveset A, Richardson DM. Mutualistic Interactions and Biological Invasions. Annual Review of Ecology, Evolution, and Systematics, Vol 45. 2014;45:89–+.

[4] Bartomeus I, Vilá M, Steffan-Dewenter I. Combined effects of Impatiens glandulifera invasion and landscape structure on native plant pollination. Journal of Ecology. 2010;98(2):440–50.

[5] Campbell C, Yang SA, Albert R, Shea K. Plant-pollinator community network response to species invasion depends on both invader and community characteristics. Oikos. 2015;124(4):406–13.

[6] Traveset A, Heleno R, Chamorro S, Vargas P, McMullen CK, Castro-Urgal R, et al Invaders of pollination networks in the Galapagos Islands: emergence of novel communities. Proceedings of the Royal Society B-Biological Sciences. 2013;280(1758). 10.1098/rspb.2012.3040 PMC3619457 23486435

[7] Tiedeken EJ, Stout JC. Insect-Flower Interaction Network Structure Is Resilient to a Temporary Pulse of Floral Resources from Invasive Rhododendron ponticum. PLoS One. 2015;10(3). 10.1371/journal.pone.0119733 PMC4357452 25764085

[8] Vilá M, Bartomeus I, Dietzsch AC, Petanidou T, Steffan-Dewenter I, Stout JC, et al Invasive plant integration into native plant-pollinator networks across Europe. Proc Biol Sci. 2009;276(1674):3887–93. 10.1098/rspb.2009.1076 19692403 PMC2817287

[9] Waser NM, Chittka L, Price MV, Williams NM, Ollerton J. Generalization in pollination systems, and why it matters. Ecology. 1996;77(4):1043–60.

[10] Ramos-Jiliberto R, Valdovinos FS, de Espanes PM, Flores JD. Topological plasticity increases robustness of mutualistic networks. Journal of Animal Ecology. 2012;81(4):896–904. 10.1111/j.1365-2656.2012.01960.x 22313043

[11] Iler AM, Goodell K. Relative floral density of an invasive plant affects pollinator foraging behaviour on a native plant. Journal of Pollination Ecology. 2014;13:174–83.

[12] Dietzsch AC, Stanley DA, Stout JC. Relative abundance of an invasive alien plant affects native pollination processes. Oecologia. 2011;167(2):469–79. 10.1007/s00442-011-1987-z 21484398

[13] Flanagan RJ, Mitchell RJ, Karron JD. Increased relative abundance of an invasive competitor for pollination, Lythrum salicaria, reduces seed number in Mimulus ringens. Oecologia. 2010;164(2):445–54. 10.1007/s00442-010-1693-2 20585807

[14] Rathcke B. Competition and facilitation among plants for pollination In: Real L, editor. Pollination Biology. Orlando, FL: Academic Press, Inc.; 1983 p. 305–29.

[15] Waser NM. Interspecific pollen transfer and competition between co-occurring plant species. Oecologia. 1978;36:223–36. 28309130 10.1007/BF00349811

[16] Albrecht M, Padron B, Bartomeus I, Traveset A. Consequences of plant invasions on compartmentalization and species' roles in plant-pollinator networks. Proceedings of the Royal Society B-Biological Sciences. 2014;281(1788). 10.1098/rspb.2014.0773 PMC4083793 24943368

[17] Russo L, Memmott J, Montoya D, Shea K, Buckley YM. Patterns of introduced species interactions affect multiple aspects of network structure in plant-pollinator communities. Ecology. 2014;95(10):2953–63.

[18] Zurbuchen A, Landert L, Klaiber J, Müller A, Hein S, Dorn S. Maximum foraging ranges in solitary bees: only few individuals have the capability to cover long foraging distances. Biological Conservation. 2010;143(3):669–76.

[19] Greenleaf SS, Williams NM, Winfree R, Kremen C. Bee foraging ranges and their relationship to body size. Oecologia. 2007;153(3):589–96. 17483965 10.1007/s00442-007-0752-9

[20] Kearns CA, Inouye DW. Techniques for pollination biologists Niwot, Colorado: University Press of Colorado; 1993. 583 p.

[21] Bosch J, Gonzalez AMM, Rodrigo A, Navarro D. Plant-pollinator networks: adding the pollinator's perspective. Ecology Letters. 2009;12(5):409–19. 10.1111/j.1461-0248.2009.01296.x 19379135

[22] Milliken GA, Johnson DE. Analysis of Messy Data. New York, NY: Chapman and Hall/CRC; 2002. 605 p.

[23] Dormann CF, Frund J, Bluthgen N, Gruber B. Indices, graphs and null models: analyzing bipartite networks. Open Ecology Journal. 2009;2:7–24.

[24] Dormann CF. How to be a specialist? Quantifying specialisation in pollination networks. Network Biology. 2011;1(1):1–20.

[25] Spiesman BJ, Inouye BD. Habitat loss alters the architecture of plant-pollinator interaction networks. Ecology. 2013;94(12):2688–96. 24597216 10.1890/13-0977.1

[26] Guimera R, Amaral LAN. Functional cartography of complex metabolic networks. Nature. 2005;433(7028):895–900. 15729348 10.1038/nature03288 PMC2175124

[27] Dupont YL, Olesen JM. Stability of modular structure in temporal cumulative plant-flower-visitor networks. Ecological Complexity. 2012;11:84–90.

[28] Dupont YL, Olesen JM. Ecological modules and roles of species in heathland plant-insect flower visitor networks. Journal of Animal Ecology. 2009;78(2):346–53. 10.1111/j.1365-2656.2008.01501.x 19021779

[29] Guimera R, Sales-Pardo M, Amaral LAN. Classes of complex networks defined by role-to-role connectivity profiles. Nat Phys. 2007;3(1):63–9. 18618010 10.1038/nphys489 PMC2447920

[30] Gonzalez AMM, Dalsgaard B, Olesen JM. Centrality measures and the importance of generalist species in pollination networks. Ecological Complexity. 2010;7(1):36–43.

[31] Olesen JM, Bascompte J, Dupont YL, Jordano P. The modularity of pollination networks. Proceedings of the National Academy of Sciences of the United States of America. 2007;104(50):19891–6. 18056808 10.1073/pnas.0706375104 PMC2148393

[32] Thebault E, Fontaine C. Stability of ecological communities and the architecture of mutualistic and trophic networks. Science. 2010;329(5993):853–6. 10.1126/science.1188321 20705861

[33] Tylianakis JM, Laliberte E, Nielsen A, Bascompte J. Conservation of species interaction networks. Biological Conservation. 2010;143(10):2270–9.

[34] Valdovinos FS, Ramos-Jiliberto R, Flores JD, Espinoza C, Lopez G. Structure and dynamics of pollination networks: the role of alien plants. Oikos. 2009;118(8):1190–200.

[35] Skinner K, Smith L, Rice P. Using noxious weed lists to prioritize targets for developing weed management strategies. Weed Science. 2000;48(5):640–4.

[36] Stouffer DB, Cirtwill AR, Bascompte J. How exotic plants integrate into pollination networks. Journal of Ecology. 2014;102(6):1442–50. 25558089 10.1111/1365-2745.12310 PMC4277853

[37] Campbell C, Yang S, Shea K, Albert R. Topology of plant-pollinator networks that are vulnerable to collapse from species extinction. Physical Review E. 2012;86(2). 10.1103/PhysRevE.86.021924 23005802

[38] Briggs HM, Anderson LM, Atalla LM, Delva AM, Dobbs EK, Brosi BJ. Heterospecific pollen deposition in Delphinium barbeyi: linking stigmatic pollen loads to reproductive output in the field. Annals of Botany. 2016;117(2):341–7. 10.1093/aob/mcv175 26658101 PMC4724048

