# Supplementary material for: Exotic Plant Infestation Is Associated with Decreased Modularity and Increased Numbers of Connectors in Mixed-Grass Prairie Pollination Networks
Source: PLoS One. 2016 May 16;11(5):e0155068. doi: 10.1371/journal.pone.0155068 (PMC4868282; doi:10.1371/journal.pone.0155068)
Supplement: S1 Appendix — Flower species did not separate with respect to C. arvense infestation. No structure was identified in insect communities. (DOCX) [file pone.0155068.s001.docx]

**S1 Appendix.** **Nonmetric multidimensional scaling analyses for flower and insect communities on infested and non-infested plots.** Flower species did not separate with respect to *C. arvense* infestation. No structure was identified in insect communities.

**
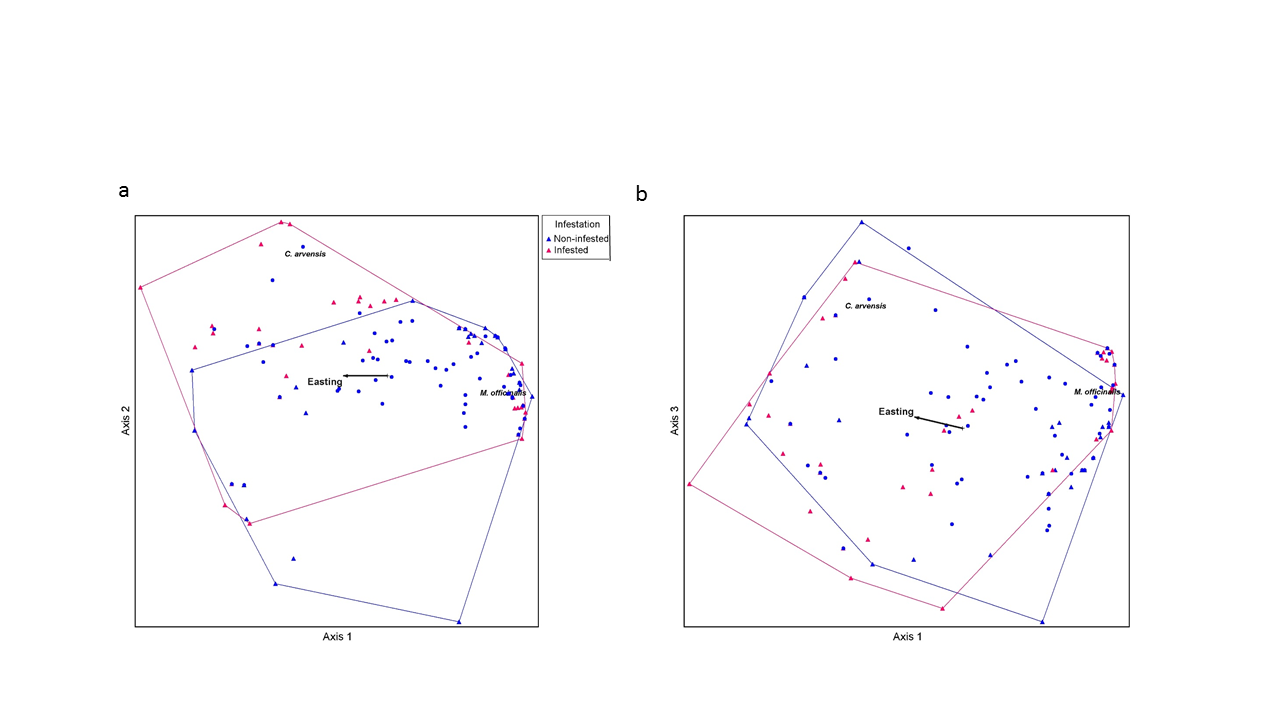
**
**S2 Fig. Plot of nonmetric multidimensional scaling analysis for flower species counts (excluding *C. arvense*) on plots at four sample periods.**  Triangles are plots, dots are flower species. For the flower matrix, three axes accounted for 78% of the variance as follows:

R Squared

Axis Increment Cumulative

1 .450 .450

2 .207 .657

3 .120 .778

Final stress for 3-dimensional solution = 9.3; number of entities = 63; number of pairs = 1953.

The plots did not separate by infestation category on any of the axes (S2 Fig), which suggests that the floral community has not diverged across our study sites based on infestation by *C. arvense*. The second axis was weakly correlated with day-of-year (a measure of progress through the season) and the first axis was correlated with Eastings (a measure of trend in geographical variation).

Axis: 1 2 3

r r-sq tau r r-sq tau r r-sq tau

Day -.219 .048 -.082 -.432 .187 -.259 -.282 .097 -.157

Easting -.469 .220 -.279 -.026 .001 .027 .231 .053 .190

No structure was detected in the community of insects that carried pollen:

R Squared

Axis Increment Cumulative

1 .019 .019
