# Supplementary material for: Exotic Plant Infestation Is Associated with Decreased Modularity and Increased Numbers of Connectors in Mixed-Grass Prairie Pollination Networks
Source: PLoS One. 2016 May 16;11(5):e0155068. doi: 10.1371/journal.pone.0155068 (PMC4868282; doi:10.1371/journal.pone.0155068)
Supplement: S1 Fig — Shown are least square means and their standard errors. Differences between infested and non-infested plots were not statistically significant at any sample period. (DOCX) [file pone.0155068.s002.docx]

**S1 Fig. Mean number of flowers of all species counted per sample period on non-infested and infested plots.**  Shown are least square means and their standard errors. Differences between infested and non-infested plots were not statistically significant at any sample period.
