# Supplementary material for: Exotic Plant Infestation Is Associated with Decreased Modularity and Increased Numbers of Connectors in Mixed-Grass Prairie Pollination Networks
Source: PLoS One. 2016 May 16;11(5):e0155068. doi: 10.1371/journal.pone.0155068 (PMC4868282; doi:10.1371/journal.pone.0155068)
Supplement: S1 Table — Thistle flowers were removed between the third and fourth sample periods on the removal plots, but flowers had senesced on the non-removal plots concurrently, so the treatment could not be compared with nontreatment. In addition, some plots that appeared to be infested at the beginning of the study largely failed to flower, further disrupting the planned design. (DOCX) [file pone.0155068.s003.docx]

**S1 Table. Mean *C. arvense* flower counts over the four sampling periods on non-removal and removal plots.** Thistle flowers were removed between the third and fourth sample periods on the removal plots, but flowers had senesced on the non-removal plots concurrently, so the treatment could not be compared with non-treatment. In addition, some plots that appeared to be infested at the beginning of the study largely failed to flower, further disrupting the planned design.

|  | **Non-removal** | | | **Removal** |  |  |  |  |  | **Mean for all plots** |
| --- | --- | --- | --- | --- | --- | --- | --- | --- | --- | --- |
| **Sample period** | **BC3** | **BP3** | **MB1** | **BC1** | **BC2** | **BP1** | **BP2** | **MB2** | **MB3** |  |
| 1 | 1 | 0 | 1351 | 145 | 8 | 12 | 5 | 7 | 76 | 176 |
| 2 | 5 | 3 | 3040 | 15 | 30 | 52 | 1 | 36 | 189 | 514 |
| 3 | 114 | 4 | 1784 | 30 | 24 | 168 | 0 | 169 | 812 | 388 |
| 4 | 0 | 3 | 0 | 0 | 13 | 0 | 0 | 86 | 0 | 34 |
